# Altered Regional Cerebral Blood Perfusion in Mild Cognitive Impairment Patients with Dizziness

**DOI:** 10.3390/diagnostics10100777

**Published:** 2020-09-30

**Authors:** Seunghee Na, Jooyeon Jamie Im, Hyeonseok Jeong, Eek-Sung Lee, Tae-Kyeong Lee, Yong-An Chung, In-Uk Song

**Affiliations:** 1 Department of Neurology, Incheon St. Mary’s Hospital, The Catholic University of Korea, Seoul 21431, Korea; seunghee.na@gmail.com; 2 Department of Nuclear Medicine, Incheon St. Mary’s Hospital, The Catholic University of Korea, Seoul 21431, Korea; jooyeon.j.im@gmail.com (J.J.I.); hsjeong@catholic.ac.kr (H.J.); 3 Department of Radiology, Incheon St. Mary’s Hospital, The Catholic University of Korea, Seoul 21431, Korea; 4 Department of Neurology, Soonchunhyang University College of Medicine, Bucheon 14584, Korea; eeksung@gmail.com (E.-S.L.); xorudoc@schmc.ac.kr (T.-K.L.)

**Keywords:** dizziness, mild cognitive impairment, single photon emission computed tomography, brain perfusion, regional cerebral blood flow

## Abstract

Dizziness is a common symptom among the general population, especially in the elderly. Previous studies have reported that dizziness may be associated with various cognitive functions including memory impairment. However, few studies have investigated the neural correlates of dizziness in patients with cognitive impairment. The aim of this study was to examine regional cerebral blood flow (rCBF) in mild cognitive impairment (MCI) patients with or without dizziness using single photon emission computed tomography (SPECT). A total of 50 patients with MCI were recruited. All participants underwent technetium-99m ethyl cysteinate dimer brain SPECT and a neuropsychological battery and completed the Dizziness Handicap Inventory (DHI). Participants were divided into a dizziness group (DHI ≥ 1, *n* = 18) and a non-dizziness group (DHI = 0, *n* = 32). Voxel wise differences in rCBF between the groups were estimated. SPECT analysis revealed decreased rCBF in the left superior temporal gyrus, left lateral orbital gyrus, and right middle frontal gyrus in the dizziness group compared with the non-dizziness group (*p* < 0.005). No significant clusters of increased rCBF were observed in the dizziness group compared with the non-dizziness group. Results of the neuropsychological tests showed a significant difference in Controlled Oral Word Association Test performance between MCI patients with and without dizziness. In conclusion, MCI patients with dizziness showed multifocal frontal and left temporal hypoperfusion compared with patients without dizziness. Our results suggest that hypoperfusion in the frontal and temporal cortices might be reflecting the negative impact of dizziness in MCI patients.

## 1. Introduction

Dizziness, the sensation of disturbed spatial orientation, is a common symptom, affecting 15%–30% of the general population [1]. Although dizziness is a broad and non-specific term with varying etiologies, it is often cited as a vestibular symptom and attributed to disorders of the vestibular system. The vestibular system is important for maintaining balance, controlling eye and head movements, and ultimately preserving a stable view of the external environment [2]. However, increasing evidence suggests that the vestibular system is involved in far more than reflex functions [3]. Findings from a national representative survey in the US indicated that vertigo is associated with increased risk of cognitive dysfunction [4]. Moreover, numerous studies in animals and humans have demonstrated that the vestibular system is linked to a variety of cognitive functions, including visuospatial ability, attention, memory, and executive function [5].

The impact of dizziness on cognitive function has been demonstrated in studies using various cognitive tasks and structural and functional neuroimaging [6,7,8,9]. Spatial memory dysfunction and disorientation were observed in patients with unilateral vestibular dysfunction, bilateral vestibular failure, and persistent postural-perceptual dizziness (PPPD) [6,7,9]. In addition to spatial cognition, non-spatial functions such as executive function, memory, and attention were affected by bilateral vestibulopathy [8,9]. Likewise, it was reported that patients with cognitive impairment, such as Alzheimer’s disease (AD) and mild cognitive impairment (MCI), have a higher prevalence of vestibular impairment [10]. Poor balance performance was found among patients with subjective cognitive decline, MCI, and dementia and the worst performance was found in patients with the most severe cognitive impairment [11]. Moreover, vestibular deficit was associated with greater decline in spatial cognition in patients with AD [10]. Interestingly, vestibular rehabilitation improved both dizziness severity and cognitive function in patients with intractable dizziness [12]. Furthermore, previous neuroimaging studies have shown alterations in brain metabolism, brain connectivity, and cortical thickness in areas involved in cognitive function among patients with various vestibular disorders, including chronic vestibular failure, bilateral vestibular failure, and PPPD [13,14,15,16,17,18,19].

Single photon emission tomography (SPECT) is a functional imaging technique that allows assessment of regional cerebral perfusion, which is often interpreted as an indirect measure of brain activity because altered neuronal activity is accompanied by corresponding changes in brain perfusion [20]. Brain perfusion SPECT has been widely used to examine brain function in various neurological and psychiatric disorders [21]. Notably, many studies have found brain perfusion correlates of cognitive dysfunction among patients with AD and MCI using SPECT [22,23]. As patients with MCI are at risk of developing dementia, physicians are particularly attentive to several MCI-related factors such as hearing impairment, co-existing mood problems, and poor balance control, which can worsen cognitive dysfunction or accelerate disease progression. Although it has been demonstrated that dizziness has a negative impact on cognition, the mechanism of the association between dizziness and cognitive impairment is still unclear. Few studies have suggested that vestibular dysfunction may lead to atrophy in cortical vestibular areas, including the hippocampus, which may in turn account for the deterioration of memory and visuospatial ability [13,24,25]. However, there is a paucity of research investigating the neural correlates of dizziness in patients with cognitive impairment. Thus, the aims of this study were to compare the regional cerebral perfusion and cognitive functions between MCI patients with dizziness and those without dizziness using SPECT and neuropsychological assessment, respectively. We hypothesized that dizziness may alter regional cerebral perfusion in the multisensory vestibular and frontal cortices and also negatively affect frontal/executive functions in MCI patients.

## 2. Materials and Methods

### 2.1. Participants

Inclusion criteria were age ≥45, right-handed, Clinical Dementia Rating (CDR) score of 0.5, and meeting the criteria for MCI, as defined by Petersen [26], which includes (a) memory complaints reported by an informant, (b) objective memory impairment for age and education, (d) largely intact functional daily activities, and (e) not demented. Exclusion criteria were any active vestibular disorders such as benign paroxysmal postural vertigo, acute vestibulopathy, and Ménière’s disease, Axis I psychiatric disorders, prominent extrapyramidal symptoms due to drugs or Parkinson’s disease, structural brain lesions (e.g., tumor or symptomatic stroke), or hearing impairment that cannot be corrected with hearing aids (Table 1). Participants who scored 1 or higher on the Dizziness Handicap Inventory (DHI) were classified as the dizziness group and those who scored 0 were classified as the non-dizziness group. This study was approved by the Institutional Review Board of the Incheon St. Mary’s Hospital (OC18ENSI0102, approved on 24 December 2018) and carried out in accordance with the Declaration of Helsinki.

### 2.2. Clinical Assessment

Clinical assessment consisted of demographics, medical history, a neurological examination, and a neuropsychological battery. Dementia severity was assessed using the CDR scale. Cognitive function was assessed using the Mini-Mental State Examination (MMSE) [27] and the presence of depressive symptoms was assessed with the Geriatric Depression Scale–Short Form (GDS-SF) [28]. Patients underwent a neuropsychological battery, the Seoul Neuropsychological Screening Battery-II (SNSB-II) [29], which consists of various cognitive tasks including the Digit Span Forward for attention domain, the Korean version of the Boston Naming Test (K-BNT) for language domain, the Rey Complex Figure Test (RCFT) for visuospatial and memory domains, the Seoul Verbal Learning Test for memory domain, and the Controlled Oral Word Association Test (COWAT) and the Stroop Test for frontal/executive domain. Dizziness severity was measured with the 25-item Dizziness Handicap Inventory (DHI) [30], giving a total score (range: 0–100) that indicates the self-perceived level of handicap associated with dizziness.

### 2.3. SPECT Acquisition and Analysis

Brain SPECT was performed using a dual-headed gamma camera (Discovery NM630; GE Healthcare, Milwaukee, WI, USA) equipped with a low-energy fan-beam collimator. Images were obtained 40 min after intravenous injection of 555–740 MBq of technetium-99m ethyl cysteinate dimer (Tc-99m ECD). Images were taken by rotating the camera a total of 720° at 6 degree intervals at a rate of 12 s per frame. Continuous transaxial brain images were reconstructed in a 128 × 128 matrix with a pixel size of 1.95 × 1.95 mm (field of view = 250 mm, slice thickness = 2.08 mm) and a 20% symmetric energy window at 140 keV using the ordered-subset expectation maximization (OSEM) algorithm (6 iterations and 10 subsets) and a Butterworth filter (cutoff frequency of 0.5 cycles/pixel and power of 10.0) to reduce noise.

Image preprocessing and analysis were performed using Statistical Parametric Mapping 12 (SPM; The Wellcome Trust Centre for Neuroimaging, London, UK). All SPECT images were spatially normalized to the SPM SPECT template (Montreal Neurological Institute, McGill University, Montreal, Canada), re-sliced with a voxel size of 2.0 × 2.0 × 2.0 mm, and smoothed with a 16 mm full width half maximum Gaussian kernel. The relative tracer activity at each voxel was estimated as a ratio to the global mean uptake using proportional scaling. For voxel wise analysis, the two sample t-test was used to assess regional cerebral blood flow (rCBF) differences between the dizziness and non-dizziness groups, controlling for the effects of age and sex. The voxel wise significance threshold was set at *p* < 0.005 (uncorrected) with a minimum cluster size of 100 contiguous voxels.

### 2.4. Statistical Analyses

The independent t-test and chi-square test were used to compare the differences in demographic and clinical characteristics between the groups. The results of neuropsychological tests were analyzed using the Mann-Whitney test or t-test for skewed or normal distributions, respectively. Data normality was tested using the Shapiro-Wilk test. The significance level was set at *p* < 0.05 (two-tailed). All statistical analyses were performed using STATA 13 (Stata Corp., College Station, TX, USA).

## 3. Results

### 3.1. Demographic and Clinical Characteristics

A total of 50 patients with MCI were recruited and classified into either the dizziness group (*n* = 18) or the non-dizziness group (*n* = 32). The demographic and clinical characteristics of the dizziness and non-dizziness groups are summarized in Table 2. There were no significant differences between the two groups in terms of age (*p* = 0.11), sex (*p* = 0.64), years of education (*p* = 0.53), MMSE score (*p* = 0.89), GDS-SF score (*p* = 0.10), and NPI score (*p* = 0.12). The frequencies of comorbidities including hypertension, diabetes, and dyslipidemia were not significantly different between the two groups. There were also no group differences in several dizziness-associated factors including migraine, observed hearing loss, and use of hypnotics or anxiolytic drugs. In the dizziness group, patients had a mean DHI score of 16.3 ± 14.6 and 10 patients (55.6%) reported triggering events such as vestibular neuritis (1 patient, 5.6%), benign paroxysmal postural vertigo (1 patient, 5.6%), persistent perceptual-postural dizziness (5 patients, 27.8%), orthostatic intolerance (2 patients, 11.1%), and medications (1 patient, 5.6%). Neuropsychological evaluations revealed a significant difference in COWAT-phonemic test results between MCI patients with dizziness and without dizziness (9.7 ± 9.3 vs. 14.8 ± 9.1, *p* = 0.04). Other cognitive tasks of attention, language, visuospatial, and memory domains did not differ significantly between the two groups (Table 3).

### 3.2. SPECT Results

**Figure 1:**
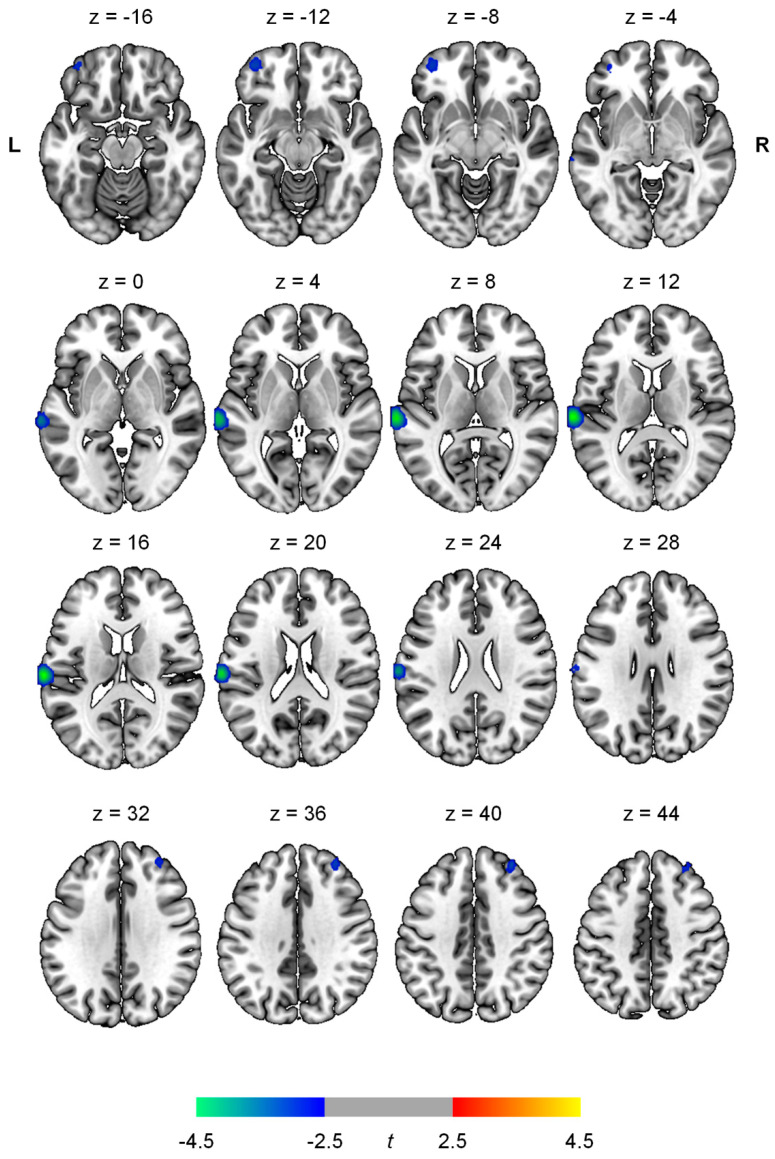
Group differences in relative regional cerebral blood flow (rCBF) between MCI patients with and without dizziness. Decreased rCBF is shown in the left superior temporal gyrus, left lateral orbital gyrus, and right middle frontal gyrus in patients with dizziness compared with patients without dizziness, after adjusting for age and sex (*p* < 0.005, extent = 100). The numbers above the brain slices indicate z coordinates in the Montreal Neurological Institute space. The color bar represents the voxel-level *t*-values.

SPECT analysis revealed decreased rCBF in the left superior temporal gyrus (peak t = 4.70, peak *p* < 0.001, peak coordinates = −68, −22, 16, cluster size = 659 voxels), left lateral orbital gyrus (peak t = 3.29, peak *p* = 0.001, peak coordinates = −40, 52, −16, cluster size = 130 voxels), and right middle frontal gyrus (peak t = 3.16, peak *p* = 0.001, peak coordinates = 34, 46, 40, cluster size = 114 voxels) in MCI patients with dizziness compared with those without dizziness (Figure 1, Table 4). There were no significant clusters of increased rCBF in MCI patients with dizziness compared with those without dizziness.

## 4. Discussion

This study used SPECT to investigate cerebral perfusion in MCI patients who did or did not have dizziness. Our results demonstrated that MCI patients with dizziness showed decreased regional cerebral perfusion in the multifocal frontal and left superior temporal cortices compared with patients without dizziness. Additionally, MCI patients with dizziness had significantly lower COWAT scores than patients without dizziness.

Previous studies have suggested that having dizziness is related to subjective memory complaints or cognitive dysfunction in various domains including visuospatial, memory, and executive function [4,6,13,31,32]. This phenomenon was also observed in patients with AD-related cognitive impairment [10,33]. In particular, vestibular dysfunction and impaired vestibular balance control were more frequently reported in patients with MCI or Alzheimer’s dementia. In this study, we found that MCI patients with dizziness performed significantly worse on the COWAT, one of the tests assessing verbal fluency and executive function, than MCI patients without dizziness, which is consistent with previous studies that showed executive function impairment among patients with dizziness [10,31]. Notably, frontal areas, especially the lateral pars orbitalis, were found to be associated with the letter-based phonemic COWAT among AD patients [34].

We also found that MCI patients with dizziness showed decreased rCBF in the left superior temporal cortex compared with patients without dizziness, which is consistent with previously reported anatomical and functional deficits observed in patients with vestibular dysfunction. The superior temporal gyrus is a component of the multisensory vestibular-sensory network [35] and it was reported that caloric vestibular stimulation induced a focal activation of regional cerebral perfusion in the bilateral superior temporal area near the auditory cortex [36,37]. In addition to vestibular input, the parieto-insula vestibular cortex (PIVC) receives proprioceptive input and visual cues and it integrates information to represent body movement and establish spatial orientation. The PIVC also plays a role in egocentric mental transformation of vestibular stimuli [38,39]. Hence, inappropriate vestibular perception or prolonged high-cortical maladaptation to the vestibular stimuli can induce anatomical and functional alterations. Moreover, the left superior temporal cortex is a substrate of auditory processing and speech comprehension [40]. Although there is dominance of the right hemisphere in multisensory vestibular cortex, several reports have demonstrated anatomical or functional changes in the superior temporal cortex and the human analog of PIVC either in the left hemisphere only or bilaterally. In patients with PPPD, a type of functional dizziness, neuroimaging studies have revealed a reduction in gray matter volume [16] and cortical folding [19] in the left superior gyrus and a decrease in connectivity between the bilateral temporal lobes and the bilateral hippocampi and parahippocampal gyri [17].

Our study also found that MCI patients with dizziness showed hypoperfusion in the left lateral orbital and right middle frontal cortices compared with patients without dizziness. Our findings are in line with findings from previous studies of altered connectivity concerning the frontal cortices and decreased rCBF in multifocal frontal areas in patients with functional dizziness [14,17,18]. These changes are expected to be consequences of compensation for dizziness as patients use their reserve or a maladaptation toward a weighting of sensory inputs other than vestibular stimuli. The right middle frontal gyrus is involved in episodic memory retrieval and has modifying activities on other brain regions [41,42]. A previous study showed that the right middle frontal gyrus volume was negatively correlated with activity in the parahippocampus and the anterior cingulate cortex, which were less activated during better performances of item memory and spatial context accuracy in older adults [42]. The orbitofrontal cortex plays an important role in choice process, which is based on the reward value of stimuli and responses [43]. A study using the Posner covert orienting task demonstrated that the lateral orbitofrontal cortex acts as a substrate for making correct responses while suppressing invalid cues, including spatial and temporal cues. Thus, dizziness, the sensation of disturbed or impaired spatial orientation [44], might cause difficulty in detecting and inhibiting external cues.

We found a significant interaction between dizziness and functional changes measured by neuropsychological tests and SPECT in patients with MCI. There were no major differences between the dizziness and non-dizziness groups with regards to confounding factors that could influence the obtained results such as migraine, observed hearing loss, or use of hypnotics/anxiolytics. Although the mechanism is unclear, several hypotheses can be assumed. Vestibular dysfunction showed a considerable impact on various cognitive domains including visuospatial, executive, and memory function [5]. These findings may be due to the anatomical and functional connectivities of the central vestibular system at the higher-level cortical areas, consisting of multisensory cortical areas connected with multiple regions of the frontal lobe, temporoparietal regions, multiple thalamic regions, and the hippocampus [45,46]. Moreover, patients with decreased brain function related to the multisensory vestibular network may have a much lower threshold for feeling dizzy compared with those with relatively preserved function. In patients with cognitive decline or the elderly, dizziness may induce the depletion of the cognitive reserve to maintain spatial orientation and balance. Reduction in cognitive reserve or neural resources available for cognitive processes was also found in patients with hearing impairment, possibly to compensate for hearing loss, although there was no difference in frequencies of observed hearing loss between the groups in our study. Furthermore, since psychiatric problems such as depression or anxiety are commonly found in both patients with dizziness and patients with MCI, psychiatric distress may be a mediator between dizziness and altered brain perfusion or cognitive function in MCI patients [47,48]. However, there are also reports that show alterations in connectivities centered in multisensory vestibular areas including hippocampi associated with vestibular dysfunction and functional dizziness regardless of psychiatric symptoms [5,17].

There are several limitations that should be considered when interpreting our results. First, this study was designed as a cross-sectional study; thus, causality cannot be confirmed between altered regional cerebral perfusion and dizziness in MCI patients. Second, the generalization of our results is limited because the etiologies and neuropathology of MCI are heterogeneous. Third, we did not perform in-depth analyses accounting for some of the potential confounding factors including underlying disease, dizziness-related factors, and use of drugs. Moreover, several factors that can influence the obtained results such as duration of dizziness, anxiety, or laboratory findings of vestibular function were not measured. To elucidate the presumed mechanism, the effects of potential confounding factors must be thoroughly considered in future studies.

In conclusion, MCI patients with dizziness showed decreased rCBF in the left superior temporal and multifocal frontal cortices, which are related to multisensory vestibular areas and executive function. Additionally, MCI patients with dizziness showed significantly poorer performance in executive function as measured by phonemic COWAT. These results suggest that dizziness may negatively affect brain function and these impacts can be revealed through the altered brain perfusion or differences of cognitive function tasks. Future studies are needed to examine whether treating dizziness symptoms can have beneficial effects for MCI patients.

## Figures and Tables

**Table 1:** Inclusion and exclusion criteria.

Inclusion Criteria
Right handed Age ≥ 45 CDR = 0.5 Petersen’s criteria for mild cognitive impairment (MCI) (a) memory complaints reported by an informant (b) objective memory impairment for age and education (c) preserved general cognitive function (d) largely intact functional daily activities (e) not demented
**Exclusion Criteria**
Active vestibular disorders such as benign paroxysmal postural vertigo, acute vestibulopathy, and Ménière’s disease Axis I psychiatric disorders Having prominent extrapyramidal symptoms due to drugs or Parkinson’s disease Having any structural brain lesions (e.g., tumor or symptomatic stroke) Hearing impairment that cannot be corrected with hearing aids

**Table 2:** Participant demographic and clinical characteristics.

Characteristics	Dizziness Group (*n* = 18)	Non-Dizziness Group (*n* = 32)	*p*
Age (years)	78.3 ± 4.8	74.1 ± 10.3	0.11
Sex (male:female)	5:13	7:25	0.64
Education (years)	5.8 ± 5.0	6.7 ± 4.4	0.53
MMSE	23.3 ± 4.0	23.5 ± 3.9	0.89
GDS-SF	8.2 ± 4.2	6.2 ± 4.0	0.10
NPI	4.4 ± 7.5	1.4 ± 3.1	0.18
Hypertension	14 (77.8%)	32 (56.3%)	0.24
Diabetes mellitus	8 (44.5%)	9 (28.1%)	0.13
Dyslipidemia	8 (44.5%)	12 (37.5%)	0.63
Sedative, hypnotic, or anxiolytic drugs	4 (22.2%)	5 (15.6%)	0.56
Observed hearing loss	1 (5.6%)	3 (9.4%)	0.63
Migraine	1 (5.6%)	1 (3.1%)	0.67
DHI	16.3 ± 14.6		
Precipitating events of dizziness (number, %)			
Vestibular neuritis	1 (5.6%)		
Benign paroxysmal positional vertigo	1 (5.6%)		
Orthostatic intolerance	2 (11.1%)		
Persistent postural-perceptual dizziness	5 (27.8%)		
Medications	1 (5.6%)		

Abbreviations: CDR, Clinical Dementia Rating; DHI, Dizziness Handicap Inventory; GDS-SF, Geriatric Depression Scale-Short Form; MMSE, Mini-Mental State Examination; NPI, Neuropsychiatric inventory.

**Table 3:** Neuropsychological test results.

Characteristics	Dizziness Group (*n* = 18)	Non-Dizziness Group (*n* = 32)	*p*
Digit Span Forward	3.0 ± 1.4	3.3 ± 1.0	0.91
K-BNT	33.4 ± 11.2	33.3 ± 1.7	0.98
RCFT-copy	24.3 ± 9.1	27.3 ± 8.7	0.17
SVLT-delayed recall	1.9 ± 1.7	2.2 ± 2.4	0.97
RCFT-delayed recall	5.4 ± 6.5	6.0 ± 6.3	0.48
COWAT-animal	10.9 ± 3.3	11.8 ± 4.3	0.42
COWAT-phonemic	9.7 ± 9.3	14.8 ± 9.1	0.04 *
Stroop test	51.2 ± 28.4	58.8 ± 30.2	0.40

Results of K-BNT, COWAT-animal, and Stroop test were analyzed via t-tests and the remaining were analyzed via Mann-Whitney tests. All values are shown as means ± standard deviations. Abbreviations: K-BNT, Boston Naming Test Korean version; RCFT, Rey Complex Figure Test; SVLT, Seoul Verbal Learning Test; COWAT, Controlled Oral Word Association Test. * *p* < 0.05.

**Table 4:** Group differences for regional cerebral blood flow.

Region	t	*p*	Coordinates * (x, y, z)	Cluster Size (Voxels)
Dizziness group < Non-dizziness group				
L superior temporal gyrus	4.70	<0.001	−68, −22, 16	659
L lateral orbital gyrus	3.29	0.001	−40, 52, −16	130
R middle frontal gyrus	3.16	0.001	34, 46, 40	114
Dizziness group > Non-dizziness group				
None				

* The coordinates refer to the Montreal Neurological Institute coordinate system. Abbreviations: L, left; R, right.
